# Report of the Organization of the Central Texas Medical Association

**Published:** 1886-10

**Authors:** H. L. Taylor

**Affiliations:** Secretary


### REPORT OF THE ORGANIZATION OF THE CENTRAL TEXAS MEDICAL ASSOCIATION.

AT a meeting of the Waco Medical Association, held September 28th, Dr. F. M. Pitts, Jr., a visiting brother, suggested the idea of calling a convention of the physicians residing in the towns and counties contiguous to Waco, to meet in the city of Waco, at some suitable time, for the purpose of organizing a District Medical Association.

The idea was heartily endorsed by the members present, and a committee appointed to issue a call for the convention, to be held in Waco, October 12th, at 10 a. m.

The call was issued, and, at the appointed time, a number of physicians from various places gathered in the hall of the Waco Medical Association, for the purpose above named.

On motion of Dr. H. W Brown, of Waco, Dr. D. R. Wallace, of Terrell, was called to the chair, and Dr. H. L. Taylor, of Waco, requested to act as temporary secretary.

By request of the Chairman, Dr. Brown made a few appropriate remarks, stating the object of the meeting and welcoming the visiting physicians to Waco, and extending to them the hospitality of her physicians and people.

Dr. Taylor, in behalf of the committee who issued the call for the meeting, stated that the object in inserting the clause "Auxilliary to the State Medical Association," was to dispel any belief which might arise in the minds of the profession, that the Central Texas Medical Association was intended to antagonize the Texas State Medical Association. He did not favor making this Association dependent upon, or subservient to the State Association or any other, but favored a complete independence in every particular.

Dr. H. C. Ghent, of Belton, said he heartily favored the organization of a District Medical Association, and would co-operate with it in all its movements, whether independent of, or auxilliary to, the State Association.

Dr. R. P. Talley, of Belton, said he was in favor of making the Association independent of everything, expossing all political methods and everything which did not bear directly on practical medicine. He wanted to see a strictly Medical Society, where subjects pertaining to the practical side of the profession might be discussed, and where papers on medical subjects might be analyzed and criticised by members of the profession in a friendly way.

Dr. H. L. Parsons, of Waco, said he heartily endorsed what had been said by the gentlemen who had preceded him, and, in order to further the movement in hand, he moved that the Chair appoint a committee of five on permanent organization, to report at the afternoon session.

Motion carried, and chair appointed Drs. H. L. Parsons, H. W. Brown, W. A. Howard, R. P. Talley and H. C. Ghent.

Dr. Brown then made a few remarks on organization, suggesting the establishment of a pathological museum and a library in connection with the Association. He then moved adjournment till 2:30 p. m., when the Association would meet for permanent organization.

### AFTERNOON SESSION.

The Association was called to order at 3 o'clock p. m., by Temporary Chairman Wallace.

The committee on permanent organization announced that they were ready to report, and were ordered to proceed.

Dr. H. L. Parsons, as chairman of the committee, read the report, and it was adopted as read, with but few changes. (For want of space only a synopsis of the report can be given here.)

The Association shall be known as the Central Texas Medical Association. The requirements for membership are the same as those of the Texas State Medical Association.

The officers are one President, one Vice President, one Treasurer, and one Secretary, who are to be elected by ballot, and shall serve one year.

The By-Laws provides for meetings of the Association in Waco quarterly, as follows: On the second Tuesday in January, April, July and October, at 10 o'clock, a. m.

Applications for membership shall be referred to a standing committee on credentials, who shall report on the same, and the Association shall adopt by ballot.

Subjects for discussion shall be selected by a standing committee appointed by the President.

One member shall be appointed by the President to open the discussion on each subject.

A committee of arrangements is also provided for, to which all matters of business are referred.

After the adoption of the report, the Association proceeded to elect permanent officers for the ensuing year, as follows: For President, Dr. F. M. Pitts, Jr., of Mount Calm; Vice-President, Dr. H. W. Dudley of Hillsboro; Treasurer, Dr. R. P. Talley of Belton; Secretary, Dr. H. L. Taylor of Waco.

The President then announced the following standing committees: Credentials—Drs. Ghent, Wallace and Metcalfe; Arrangements—Drs. Brown, King and Parsons; Subjects—Drs. Talley, Dudley and Barker.

The Committee on Subjects then retired to select subjects for discusion at the next regular meeting.

Moved and carried, that a copy of proceedings be sent to each of the Texas medical journals, with a request that they be published.

Numerous letters from prominent physicians in all parts of the State, endorsing the movement, and asking that they be allowed to become members, were read and the applications approved.

The Committee on Subjects reported, and the following were chosen from the report by the Association for discussion at the next meeting:

1. Syphillis when Contracted can be Eradicated; discussion to be opened by Dr. R. P. Talley of Belton.

2 Typho-Malarial Fever, So-called; discussion to be opened by Dr. W. L. Barker of Waco.

After attending to several minor matters, which it is not necessary to report, the Association adjourned to meet in Waco on the SECOND TUESDAY IN JANUARY, 1887, AT 10 O'CLOCK, A. M.

The meeting was harmonious throughout, and twenty-eight (28) members joined the Association.

The Secretary takes pleasure in extending a cordial invitation to all physicians in good standing in the State to be present at the next meeting and become members of the Association. It is not merely a local affair, but is intended for all who wish to become members.

H. L. TAYLOR, Secretary.

---

TO MEMBERS OF THE STATE MEDICAL ASSOCIATION:

The Secretary has on hand a number of copies of the Transactions for 1884 and 1885. New members, and members who failed to receive a copy will be supplied on application, enclosing 17 cents for postage.

The new volume of Transactions (1886) is now ready, and will be mailed to members post-paid. It is the handsomest volume of Transactions ever gotten out by any State Association without exception.

---

Being desirous of building up the *Physicians' Mutual Benefit Association*, and, at the same time, our subscription list, we make the following offer: Those who subscribe (and remit the price of subscription) between now and December 1st, will be given a membership in the *Physicians' Mutual Benefit Association free*, if they desire it. There will be nothing to pay then till January 1st, when all are required to pay the annual dues of $1.00 for the new year. Of course, if a death occurs meantime, all members will be assessed $1.00.

---

Convulsions may be frequently cut short like magic by turning the patient on his left side. The nausea, as an after effect of chloroform and ether narcosis, may generally be controlled in the same manner.—*Chicago Medical Times*.

